# Effects of Agent-Environment Symmetry on the Coordination Dynamics of Triadic Jumping

**DOI:** 10.3389/fpsyg.2017.00003

**Published:** 2017-02-02

**Authors:** Akifumi Kijima, Hiroyuki Shima, Motoki Okumura, Yuji Yamamoto, Michael J. Richardson

**Affiliations:** ^1^Department of Education, University of YamanashiKofu, Japan; ^2^Department of Environmental Sciences, University of YamanashiKofu, Japan; ^3^Department of Art and Sports Educational Science, Tokyo Gakugei UniversityKoganei, Japan; ^4^Research Center of Health, Physical Fitness and Sports, Nagoya UniversityNagoya, Japan; ^5^Department of Psychology, Center for Cognition, Action and Perception, University of CincinnatiCincinnati, OH, USA

**Keywords:** joint action, symmetry, symmetry-breaking, leader and follower roles, social motor coordination

## Abstract

We investigated whether the patterns of coordination that emerged during a three-participant (triadic) jumping task were defined by the symmetries of the (multi) agent-environment task space. Triads were instructed to jump around different geometrical arrangements of hoops. The symmetry of the hoop geometry was manipulated to create two symmetrical and two asymmetrical participant-hoop configurations. Video and motion tracking recordings were employed to determine the frequencies of coordination misses (collisions or failed jumps) and during 20 successful jump sequences, the jump direction chosen (clockwise vs. counterclockwise) and the patterning of between participant temporal movement lags within and across jump events. The results revealed that the (a)symmetry of the joint action workspace significantly influenced the (a)symmetry of the jump direction dynamics and, more importantly, the (a)symmetry of the between participant coordination lags. The symmetrical participant-hoop configurations resulted in smaller overall movement lags and a more spontaneous, interchangeable leader/follower relationship between participants, whereas the asymmetrical participant-hoop configurations resulted in slightly larger overall movements lags and a more explicit, persistent asymmetry in the leader/follower relationship of participants. The degree to which the patterns of behavioral coordination that emerged were consistent with the theory of symmetry groups and spontaneous and explicit symmetry-breaking are discussed.

## 1. Introduction

Suppose you oscillate the index finger of each hand back and forth at the same time. The abductors and adductors of the two fingers contract simultaneously. This pattern of synchronous coordination is commonly termed in-phase coordination and reflects a symmetric pattern of behavioral action, in that the phase or spatiotemporal position of the two movements is exactly (or nearly exactly) the same over time (they are 0° out of phase). In contrast, if you oscillate one index finger leftward and the other index finger rightward at the same time, adduction and abduction occur in an asymmetric manner. This latter pattern of behavioral synchrony is commonly termed anti-phase coordination, because the phase of the finger movements are exactly (or nearly exactly) opposite over time (they are 180° out of phase). Now, suppose you start oscillating your two index fingers in an anti-phase or asymmetric manner at a relatively slow movement frequency (say one oscillation a second or 1 Hz) and then gradual increase your movement frequency over time so that your fingers move faster and faster. What you find is that at very fast frequencies of movement your fingers will spontaneously transition from anti-phase coordination to the symmetric, in-phase pattern of coordination. In fact, this transition will likely occur no matter how hard you try to maintain an asymmetric or anti-phase pattern of coordination; the transition is indifferent to your will. Finally, try and produce a pattern of coordination between your two index fingers that is neither in-phase nor anti-phase. Like trying to maintain anti-phase coordination at fast movement frequencies, you will find that this is also nearly impossible to do, with your fingers being spontaneously pulled back into an in-phase or anti-phase pattern of movement (and more often in-phase than anti-phase). Interestingly, if you try the same experiment with your hands, your arms, your legs, or any two limbs for that matter, you will find the same result; namely, that (two-limb) rhythmic inter-limb coordination is constrained (without practice) to in-phase and anti-phase patterns of coordination, with in-phase-coordination more stable than anti-phase coordination.

This highly robust rhythmic coordination phenomenon was empirically demonstrated by Scott Kelso in the mid 1980s (Kelso, 1984, 1995) and has been effectively modeled (Haken et al., 1985) in a manner consistent with the dynamics of coupled oscillators. Of more relevance here, is that the symmetry of these two coordination patterns are defined by the symmetry of the underlying dynamics of the component limbs (oscillators) and the inter-limb coupling (Golubitsky et al., 1998, 1999). In more formal terms, in-phase coordination reflects a symmetric mode of coordination because it preserves the symmetry of the system. That is, the observed pattern of coordination is invariant to the spatial permutation or interchange of oscillator (movement) 1 and oscillator (movement) 2. In contrast, the anti-phase mode of coordination reflects a state of less or broken symmetry, in that the pattern of coordination is no longer invariant to a purely spatial permutation or interchange of the two oscillators (movements). It is important to appreciate, however, that anti-phase coordination is still very much entailed by the symmetry of the system of two identical (or near identical) oscillatory movements and does not correspond to a state of no symmetry. Rather anti phase coordination is symmetric with respect to the spatiotemporal transformation that permutes the two oscillators/movements and shifts the phase by half a period (see e.g., Collins and Stewart, 1994; Kelso, 1995; Richardson et al., 2015 for more details about the spatial and temporal symmetries of the coupled oscillators).

The importance of understanding rhythmic coordination in terms of symmetry is that the theoretical principles of symmetry and symmetry-breaking provide a lawful, yet highly generalizable understanding of behavioral coordination that is indifferent to the particulars of the system, movement, or coordination task being considered. For instance, Golubitsky and Stewart (2003) have demonstrated how the different rhythmic gait patterns observed in human, animal, and insect locomotion are a lawful consequence of the finite set or group of symmetries that define the couplings between the cells of the central pattern generators assumed to underlie gait control.

For example, the gait patterns of quadrupeds are defined by the symmetry group that includes invariance in the permutation between two contralateral cells and also includes invariance in the permutation of four ipsilateral cells (see Golubitsky et al., 1998, 1999; Buono and Golubitsky, 2001). Such symmetry predictions also provide a generalized understanding human arm-leg (4-limb) coordination (Jeka et al., 1993). Harrison and Richardson (2009) have even demonstrated how the gait patterns of two individuals walking one behind the other are spontaneously confined to patterns predicted by the symmetry group approach of Golubitsky and colleagues (see Richardson et al., 2016 for more details).

The significance of the latter interpersonal example, is that it demonstrates how symmetry principles not only define intrapersonal and biomechanically coupled patterns of movement coordination, but also appear to underlie social or informational (visually, auditory) coupled patterns of movement coordination. Perhaps the most famous example of this with regards to rhythmic coordination stems from the work of R.C. Schmidt and colleagues, which has demonstrated how the rhythmic limb or body movements of visually coupled participants are constrained to the exact same, in-phase and anti-phase patterns of coordination defined above (e.g., Schmidt et al., 1990; Amazeen et al., 1995; Schmidt and O'Brien, 1997; Richardson et al., 2005, 2007b). As with intrapersonal rhythmic coordination (Kelso, 1984, 1995), the stability of in-phase (symmetric) coordination during interpersonal or visually mediated interaction is greater than that observed for anti-phase (asymmetric) coordination, evidenced by the greater variability of anti-phase coordination compared to in-phase coordination and that visually coupled individuals spontaneously transition from anti-phase to in-phase coordination at faster movement frequencies (Schmidt et al., 1990; Schmidt and Turvey, 1994).

Note the relationship between the order of the symmetry that defines the coordination pattern and the stability of that coordination pattern, not to mention how symmetric systems will tend to exhibit more symmetric patterns of behavior if possible (Kugler and Shaw, 1990; Kelso, 1995; Turvey, 2007). The transition from more to less symmetric states is also, possible, however, if a more symmetric state of behavior becomes unstable beyond some critical control parameter value (i.e., a *spontaneous symmetry break* occurs) or if some form of asymmetry is introduced into the system (i.e., *explicit symmetry breaking* occurs). Richardson et al. (2015) have recently argued that the principle of symmetry and the theory of spontaneous and explicit symmetry-breaking provides a highly generalizable way of understanding and predicting the organization and stable patterns of human and social behavior. Motivated by Curie's principle (“the symmetry of the effects are written in the symmetry of the causes” Curie, 1894) and the theory that *symmetry breaks operate to create higher order structures of behavioral organization*, they argue that the modes or patterns of behavior exhibited by individuals during joint- or social-activity are often a result of spontaneous or explicit symmetry breaking events or task properties (also see Lagarde, 2013; Richardson et al., 2016). As evidence of this, they highlight recent research demonstrating how experimentally assigned leader/follower roles naturally induce compensatory behavioral action on the part of the leader in order to help stabilize a followers action (Vesper and Richardson, 2014). Conversely, individuals often spontaneously induce such symmetry breaks during on-going joint-action in order to establish more stable patterns of behavior. For instance, Richardson et al. (2015) observed that pairs of individuals instructed to rhythmically move back and forth between orthogonally opposed targets unexpectedly adopted an asymmetric pattern of elliptical movement in order to minimize the chance of a collision and at the same time maximize coordination stability. Moreover, the spontaneous appearance of the asymmetric movement pattern established a complementary leader/follower relationship that persisted for the remainder of the experimental task.

Recent research examining the stable patterns of real-world multi-agent behavior have revealed findings compatible with the symmetry approach. For instance, during many two person sports tasks, inter-player coordination patterns intermittently transition between two (Kijima et al., 2012; Okumura et al., 2012) or more stable states or modes of behavior (Yamamoto et al., 2013), with most of these modes reflecting an asymmetrical pattern of behavioral order (e.g., anti-phase) that is dependent on environmental or task constraints (e.g., interpersonal distance). The role of a player (e.g., step forward or away, offense or defense) alters accordingly (Kijima et al., 2012). Such role asymmetries are characteristic of the sports like soccer (Yamamoto and Yokoyama, 2011) and basketball (Fujii et al., 2016) and can depend on the skill of the players. For example, Yokoyama and Yamamoto (2011) asked four participants, including collegiate soccer players, to engage in a simplified three on one soccer game (monkey in the middle game) and found that the symmetry of the coordination patterns adopted by players were skill dependent realizations of behavioral coordination modes predicted by the symmetries of symmetric Hopf bifurcation theory (Golubitsky and Stewart, 1985). In simple terms, the coordination patterns of triads with higher skill level had higher order spatiotemporal symmetry.

## 2. Experiment

**Figure 1:**
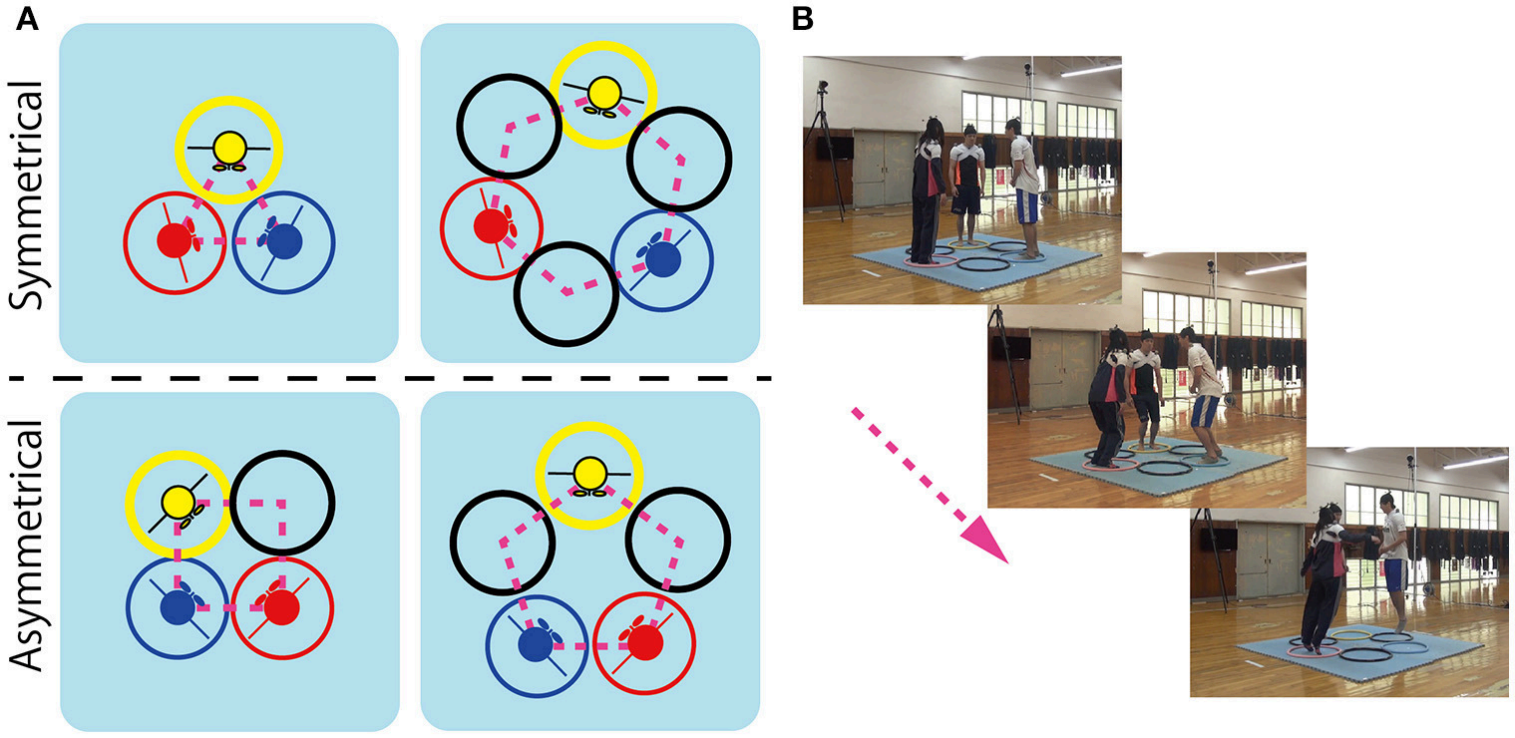
**The four hoop conditions employed for the triad jumping task as a function of the symmetry of spatial jumping degrees-of-freedoms (DoF) available for participants in a triad**. The triangle (3-hoop; top left) and hexagon (6-hoop; top right) conditions were considered *symmetric* as each participant had equivalent jumping DoF (i.e., every jumper had the same number of adjacent hoop spaces open; or not open in the case of the triangle condition). In contrast, the square (4-hoop; bottom left) and pentagon (5-hoop; bottom right) conditions were considered *asymmetric* because jumpers did not have equivalent jumping degrees of freedom; i.e., not all jumpers had the same number of adjacent hoop spaces open. **(A)** Sequence of jumping action from setup (left top), middle (middle), and jump (right bottom) **(B)**.

The aim of the current study was to further explore the degree to which the behavioral organization or patterning of social movement coordination is a consequence of the symmetry (or asymmetry) of the physical and informational constraints that define a given agent-environment task context. In other words, the current study was aimed at testing whether the (a)symmetry of a task's action space defines what (possible) patterns of social movement coordination should be observed. To achieve this aim, a three person (triad) coordinated jumping task was developed, in which participant triads were required to jump around different geometrical arrangements of hoops without colliding or bumping into each other. Four different geometric hoop arrangements were employed, 3-, 4-, 5-, and 6-hoop arrangements (see Figure 1), such that symmetry of the participant-hoop configuration was greater in the 3- and 6-hoop conditions (referred to as the symmetric conditions) compared to the 4- and 5-hoop conditions (refereed to as the asymmetric conditions).

The study centered on two related predictions. The first concerned jumping direction. Essentially, on each jump event the participants in a triad needed to all jump in the same clockwise or counterclockwise direction in order to avoid colliding. Note that clockwise and counterclockwise jumping were equally afforded in all hoop conditions. In other words, clockwise and counterclockwise jumping were symmetrically stable. Therefore, it was necessary for the participants to collectively break this symmetry on a given jump such that everyone jumped in the same direction. Given that participants were instructed not to talk or non-verbally indicate their intended jumping direction, it was expected that successful jumping sequences would result when this symmetry was spontaneously broken on the first trial and then explicitly (induced) on subsequent jumping trials. That is, *participants were expected to explicitly break the symmetry of jump direction by jumping in the same direction over the course of repeated jumping events*. However, *given the symmetric possibility of clockwise or counterclockwise jumping, both direction preferences were expected to be observed across triads (i.e., the global symmetry of clockwise or counterclockwise jumping was expected to be preserved across triads)*.

The second prediction concerned the temporal patterning of the participant's jumps, with different patterns expected for the different hoop conditions. In simple terms, the number of open hoops was the same (symmetric) for each participant in the 3-hoop (triangle) and 6-hoop (hexagon) conditions, but different (asymmetric) in the 4-hoop (square) and 5-hoop (pentagon) conditions. More formally, the symmetry of the different participant-hoop configurations can be defined by the group (set) of symmetry transformations (rotations and reflections) that resulted in the geometry of the participant-hoop arrangement remaining invariant (i.e., remaining equivalent or unchanged). Of particular importance was that the corresponding symmetry group for each participant-hoop configuration was equal to the highest order common factor (the *highest order isotropy subgroup*) of the symmetry group that define the hoop and triad arrangements independently. With regard to the symmetry of the hoop arrangement, the triangular 3-hoop arrangement for instance was invariant to rotations of 120°, 240°, 360° and reflections about the three mid-point axes that dissected each hoop. These symmetry transformations correspond to the rotational symmetry group *Z*_3_ [*Z*(0, 360), *Z*(120), *Z*(240)] and the reflection symmetry group *R*_3_, respectively, and when taken together, reflect how the symmetry of the 3-hoop triangle is defined by the dihedral group *D*_3_. The symmetry group of the other geometric hoop layouts can be similarly defined, such that the 4-hoop square condition had *D*_4_ symmetry, the 5-hoop pentagon condition had *D*_5_ symmetry, and the 6-hoop hexagon condition had *D*_6_ symmetry as represented in Table 1.

**Table 1:** **Isotropy subgroups of the actor-hoop configurations**.

**Geometry**	**Hoop symmetry**	**Actor symmetry**	**Actor-hoop symmetry (isotropy subgroup)**
Triangle	*D*_3_	*S*_3_	*D*_3_ (or *S*_3_), *Z*_3_, *D*_1_ (or *Z*_2_), *I*
Square	*D*_4_	*S*_3_	*D*_1_ (or *Z*_2_), *I*
Pentagon	*D*_5_	*S*_3_	*D*_1_ (or *Z*_2_), *I*
Hexagon	*D*_6_	*S*_3_	*D*_3_ (or *S*_3_), *Z*_3_, *D*_1_ (or *Z*_2_), *I*

With regard to the geometric symmetry of the triad, a specific set of starting hoop locations were employed (see Figure 1) such that assuming that each participant was more or less equivalent in action (jumping) capability, task understanding, motivation, etc., the three participants in each triad could be assigned (interchanged) to any of the defined starting hoop locations. Hence the symmetry of the participants (actors) with regards to assigned hoop location corresponded to the symmetry group *S*_3_, meaning that there are 3! (=6) equivalent ways the actors could be permuted with regards to assigned hoop location (i.e., [1-2-3], [1-3-2], [2-1-3], [2-3-1] [3-1-2], and [3-2-1]). Accordingly, the symmetry of the relational configuration of a triad with regards to hoop alignment corresponded to highest order isotropy subgroup of a hoop conditions *D*_*n*_ symmetry group and the permutation group *S*_*n*_. As detailed in Table 1, this corresponds to *D*_3_ for the 3-hoop (triangle) and 6-hoop (hexagon) conditions and *D*_1_ (or *Z*_2_) for the 4-hoop (square) and 5-hoop (pentagon) conditions. Note that *D*_1_ has only one rotational symmetry *Z*(0, 360) and one reflection symmetry *R*_1_, due to asymmetrical or not integer factorization of the corresponding *D*_*n*_ to *S*_3_ symmetry. *I*, an isotropy subgroup of all hoop conditions, means transformation that has only one rotational symmetry *Z*(0, 360) and does not allow any permutation. (For a relevant introductory overview of Group Theory and a detail explanation about the nature of dihedral group *D*_*n*_ and its relation to *S*_*n*_ and *Z*_*n*_, see Richardson et al., 2015, p. 238.)

It is important to appreciate the novel hypothesis being tested here; namely, that the symmetry of the temporal coordination observed between triads would be consistent with the symmetry of the isotropy subgroup that defined the participant-hoop configurations. *The general prediction was that participant-hoop configurations defined by higher order isotropy subgroups (i.e., 3 and 6 hoop conditions) would result in more symmetric patterns of temporal coordination compared to the participant-hoop configurations defined by lower order isotropy subgroups (i.e., 4 and 5 hoop conditions)*. Accordingly, we expected that the symmetry of temporal lead/lag relationship (i.e., leader/follower role) between actors would be a functional reflection of isotropy subgroup that defined the participant-hoop configuration. More specifically, we expected that the *D*_3_ hoop-triad symmetry of the 3- and 6-hoop conditions would result in a symmetric interchange of participants with regards to who led and followed (lagged) over the course of jumping trials and sequences. This is because the higher order *D*_3_ isotropy subgroup of the participant-hoop configurations for the 3- and 6-hoop conditions corresponded to a more symmetric action space for participants in these conditions. That is, each participant's spatial jumping degrees of freedom (DoF) were equivalent (symmetric) in the 3- and 6-hoop conditions. This action space symmetry was broken, however, in the square and pentagon conditions, and is formally realized by the lower order *D*_1_ isotropy subgroup of the corresponding participant-hoop configuration. Indeed, for the square and pentagon conditions the spatial jumping DoF of participants are asymmetric (see Figure 1A). For the square condition two participants have one (common) open space adjacent to them, whereas the third participant does not. For the pentagon condition, one participant has two adjacent open spaces, whereas the other two participants only have one. Accordingly, we expected a corresponding asymmetry in the role of participants with regards to who led and followed (lagged) over the course of jumping trials and sequences, with one actor tending to consistently lead and/or lag behind the other two (i.e., consistent with a *D*_1_ or *Z*_2_ pattern).

### 2.1. Materials and methods

#### 2.1.1. Participants

Twenty-seven undergraduate students from Tokyo Gakugei University and the University of Yamanashi were recruited as participants in the study. Fifteen participants were male and 12 were female, with a mean (SD) age of 20.00(±0.961) years. Participants were randomly assigned to one of nine triads. Participant handedness, or laterality quotient (H) for each participant was determined using the 10 item Edinburgh inventory of handedness (Oldfield, 1971). H value ranges from −100, which corresponds to extreme left-handedness, to +100, which corresponds to extreme right-handedness. H for one female member was −21.739, indicating weak left-handedness (≤ 1 in decile score). The mean (SD) H score for the remaining 26 participants was 60.773(±21.510), with a range of 8.33 (very weak: 1 in decile) to 100.00 (completely right-handed: 10 in decile).

#### 2.1.2. Jumping task & task space geometry

*Jumping task:* Participant triads were instructed to jump in a clockwise or counterclockwise direction around geometric arrangements of three, four, five or six 0.6 m diameter rubber hoops placed on the center of 2.28 × 2.28 *m*^2^ polyurethane mat (see Figure 1B). The hoops were aligned such that both sides of a hoop touched adjacent hoops and the distance between hoops was equal, resulting in the four geometric hoop arrangements: a 3-hoop triangle, a 4-hoop square, a 5-hoop pentagon, and a 6-hoop hexagon (see Figure 1A). Each member of the triad was assigned to one of the three colored hoops (i.e., yellow, blue, or red in Figure 1). This hoop corresponded to a participant's starting hoop location.

Each member of a triad was instructed to jump with both legs into an adjacent hoop (either to the left or right) at the sound of a specific metronome cue. Participants were instructed to jump together as a group and to avoid colliding into each other. The metronome tone was presented at 1.0 s intervals, with every third metronome beat presented at a higher tone to indicate the time to jump (i.e., the jumping movement cycle ≈ 3.0 s). Participants were informed that they should continue to jump every 3-s (i.e., every higher metronome tone) until they succeeded in performing a sequence of 20 successfully coordinated jumps. If any participant in a triad collided with another participant (i.e., performed an unsuccessfully coordinated jump), the participants were instructed to stop and move back to their assigned starting hoop location and begin the sequence again.

Four triads began with the triangle condition and the number of hoops was increased one by one when the triads completed a sequence of 20 successful trials in the given geometric condition. The remaining five triads began with the hexagon condition and the number of the hoops was decreased. No instructions were provided as to which direction participants should jump. Rather participants were informed that jumping direction could be freely selected at the time of each jump, with the understanding that each member of a triad had to jump in same direction in order to avoid collision. Participants were not informed about what lead/lag relationships should or could be employed, nor were participants designated a-priori as leader/follower (absolutely no information about possible leader/follower roles was provided to participants). Participants were also given explicit instructions not to verbally or non-verbally communicate with each other during the experiment. Accordingly, each member of a triad had to predict the other two members' jumping direction while preparing to execute their own jumping movement. In this preparation phase, downward movement of the center of mass and forward/upward arm swing would be required to recoil enough to jump the distance between the hoops (max inter-hoop jumping distance was 0.6 m).

As detailed above, the participant-hoop configurations employed in the current study were defined by the isotropy subgroups listed in Table 1. As further clarification, note that the participant (actor) symmetry, *S*_3_ was isomorphic with the symmetry group *D*_3_, which can be seen by the fact that the three actors always form a triangle within the task space (*S*_3_ and *D*_3_ are equivalent symmetry groups). With regards to hoop alignment for the two symmetrical conditions, the symmetry of the triangle and hexagon conditions are captured by the dihedral group *D*_3_ and *D*_6_, respectively. Accordingly, for both the triangle and hexagon conditions the isotropy subgroups of the hoop-participant configuration are *D*_3_, *Z*_3_, *D*_1_ (or *Z*_2_,) and *I* (identity), with the highest order subgroup being *D*_3_. For the asymmetric groups, the symmetry of the square and pentagon conditions is captured by the dihedral group *D*_4_ and *D*_5_, respectively. Thus, for both of these conditions the isotropy subgroups of the hoop-participant configuration are only *D*_1_ (or *Z*_2_,) and *I* (identity), with the highest order subgroup being *D*_1_ (*D*_1_ = *Z*_2_ are isomorphic groups and reflect the fact the system is invariant to only 2 transformation; no-change and the reflection/permutation of only two of three elements).

#### 2.1.3. Procedure

After arriving at the testing location, participants were given written informed consent in accordance with the Declaration of Helsinki. Following informed consent, each participant completed the 10-item Edinburgh inventory of handedness (Oldfield, 1971). Participants then received instructions about the jumping task and how the jumping task should be performed (again, note that no information about how the task should be completed successful was provided, either in terms of direction, lead/lag relationships, or leader/follower roles). Following these instructions, a cap with 4 motion-tracking markers attached to it was placed on each participant's head to record the jumping movements (see below for more details on the motion tracking systems and markers employed). After the marker cap was secured to each participant's head and participants indicated that they understood the task instructions, participants were then randomly assigned to one of the different colored hoops and were informed that this colored hoop would always be their starting hoop (location) during the experiment. Triads then practiced the task several times. At the beginning of each jumping sequence, each member of a triad was instructed to stand in their assigned hoop while the metronome tone was presented. Participants started jumping at the time of an experimenter's verbal cue, with the aim of completing 20 successful jumps in a row. As mentioned above, if a collision occurred at any time during a jumping sequence, participants were instructed to return to their respective starting locations and begin the jumping sequence again. The experiment ended when a triad completed 20 successful trials for all four hoop conditions. Triads performed the task alone and did not view other triads performing the task before their own performance. Members of each triad were acquainted with each other, as they were all students from the same course (physical education) at the same university (Tokyo Gakugei University or University of Yamanashi). These procedures adhered to the Faculty of Education in University of Yamanashi research ethics committee guidelines.

#### 2.1.4. Dependent measure and analysis

Six infrared cameras (OQUS300, Qualysis, Sweden) were used to record the three-dimensional position of each participant's head location at a sampling frequency of 100 Hz. Each participant wore a 4-marker head cap; three markers aligned in triangle shape to detect participant's head direction and one marker located in the center of the triangle to detect the central position of the head. Prior to analysis, the recorded motion data was filtered using fourth ordered Butterworth filter with cutoff frequency of 6 Hz. Task performance was also recorded using a digital video camera (Sony DCR650; 60 Hz) to determine (1) the frequency of unsuccessful jumps (coordination collision or misses) prior to a triad completing a 20 successful jump sequence for a given condition and (2) the direction of jump rotation, measured in terms of the frequency of counterclockwise rotation.

**Figure 2:**
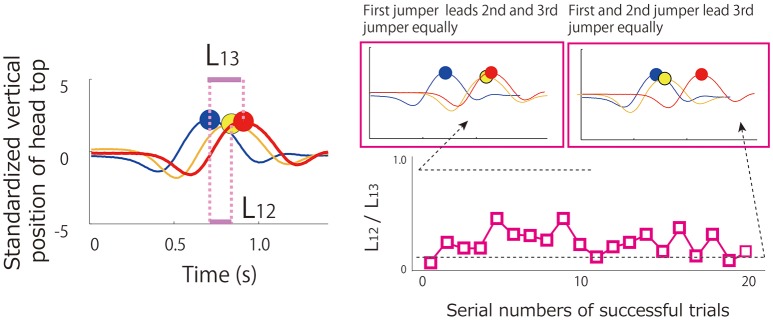
**Illustration of coordination lag and coupling pattern index calculations**. Left panel depicts hypothetical variation of vertical head position of triad. The timing of peak height is indicated by the colored circles. The lag value of *L*_13_ denotes the lag between 1st and 3rd person to jump and *L*_12_ denotes the lag between the 1st and 2nd person to jump. Coupling pattern index (CI) can be calculated by dividing *L*_12_ by *L*_13_. Hypothetical variation of CI during a 20 successful repetition jumping sequence is depicted in the right panel.

Measures indicating the participants' temporal behavioral patterns and in particular their leader/follower status were most/more relevant for our research. Especially, the measure to indicate leader/follower status would be more important in current research because the status was not a-priori assigned in the task that would be emerge dependent on participants-hoops geometrical configuration. Therefore, to determine the temporal coordination of triads, the motion data of each participant's head height (head position on the Z-axis) was first divided into each jump cycle by isolating the peaks (top most head positions) over time. From these participant head-height peaks, the lead/lag jumping time between participants at each jump was calculated with respect to the first (lead) jumper (see Figure 2). That is, at every jump event the temporal lag of the two follower jumpers was determined with respect to the lead jumper. These standardized lag times were then averaged to provide an overall estimate of the temporal coordination lag. A coupling pattern index (CI) was also calculated from these lag times and was equal to

(1) $$\begin{array}{l} CI=\frac{L_{12}}{L_{13}} \end{array}$$

where *L*_12_ denotes the lag between the leader and the second jumper, and *L*_13_ denotes the last (third) jumper's lag with respect to the leader. As can be seen from an inspection of Figure 2, a CI ratio equal or close to 0.0 indicates a coupling pattern in which two participants essentially lead one follower, whereas a CI ratio equal or closes to 1.0 indicates a coupling pattern in which one participant lead two followers. Each triads performance and rotation data from the 20 successful jump sequences were averaged separately for each of the four geometrical hoop conditions and were compared using one-way, repeated measures ANOVAs. Temporal lag and CI were not averaged over the 20 successful trial sequences and, thus, were analyzed using two-way repeated measures ANOVAs (four geometrical conditions × 20 successful trials). *Post-hoc* analysis was conducted using Benjamin-Hochberg procedure to control discovery rate.

### 2.2. Results

#### 2.2.1. Jumping direction

**Figure 3:**
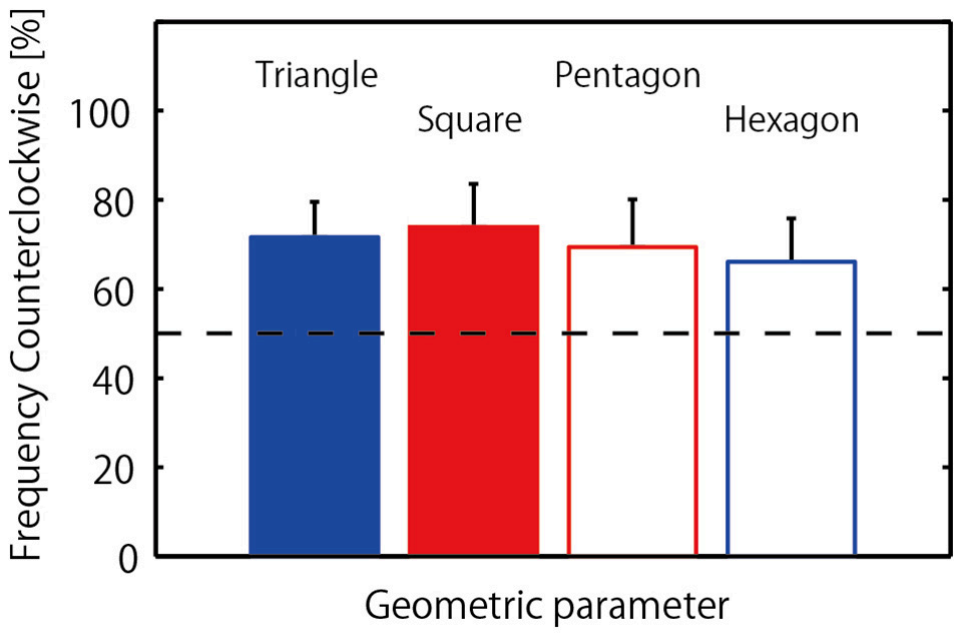
**Frequency of counterclockwise jump rotation for triads**. Horizontal broken line indicates chance level (50% for each direction).

The mean and standard deviation of frequency of counterclockwise jump rotation is displayed in Figure 3, with a one-way repeated measures ANOVA indicating no significant difference between geometrical conditions, *F*_(3, 24)_ = 0.28, *p* = 0.84, η^2^ = 0.19. Somewhat unexpectedly, during successful 20-jump sequences triads tended to jump in a counterclockwise direction in all conditions: 71.67 (±23.59)% of trials in the triangle condition; 73.89 (±29.02)% of trials in the square condition; 69.44 (±31.86)% of trials in in the pentagon condition; and 66.11 (±29.13)% of trials in for hexagon condition. To determine if this overall mean effect was representative of the triads as a whole, a binomial test was employed to confirm the significance of counterclockwise rotation frequency for each individual triad. The results indicated that the preference for counterclockwise direction was significant in 5 triads (*p* < 0.001), with two triads exhibiting a slightly over chance level of counterclockwise preference, and only one triad exhibiting a greater preference for the clockwise jump direction as demonstrated in Table 2 (Triad C: 48 clockwise jumps out of 80 successful jumps). In addition, three triads (Triad E, F, and G in Table 2) nearly always jumped in counterclockwise direction in all conditions.

**Table 2:** **Frequency of rotation direction adopted by each of nine triads (A–I)**.

**Direction**	**Triad A**		**Triad B**		**Triad C**		**Triad D**		**Triad E**		**Triad F**		**Triad G**		**Triad H**		**Triad I**	
	**c**	**w**	**c**	**w**	**c**	**w**	**c**	**w**	**c**	**w**	**c**	**w**	**c**	**w**	**c**	**w**	**c**	**w**
Triangle	12	8	9	11	9	11	10	10	20	0	20	0	20	0	15	5	14	6
Square	19	1	8	12	8	12	8	12	20	0	20	0	20	0	11	9	19	1
Pentagon	11	9	13	7	4	16	13	7	20	0	19	1	20	0	5	15	20	0
Hexagon	14	6	11	9	11	9	11	9	20	0	20	0	19	1	11	9	2	18

Direction was denoted by a letter “c (counterclockwise)” or “w (clockwise).”

**Figure 4:**
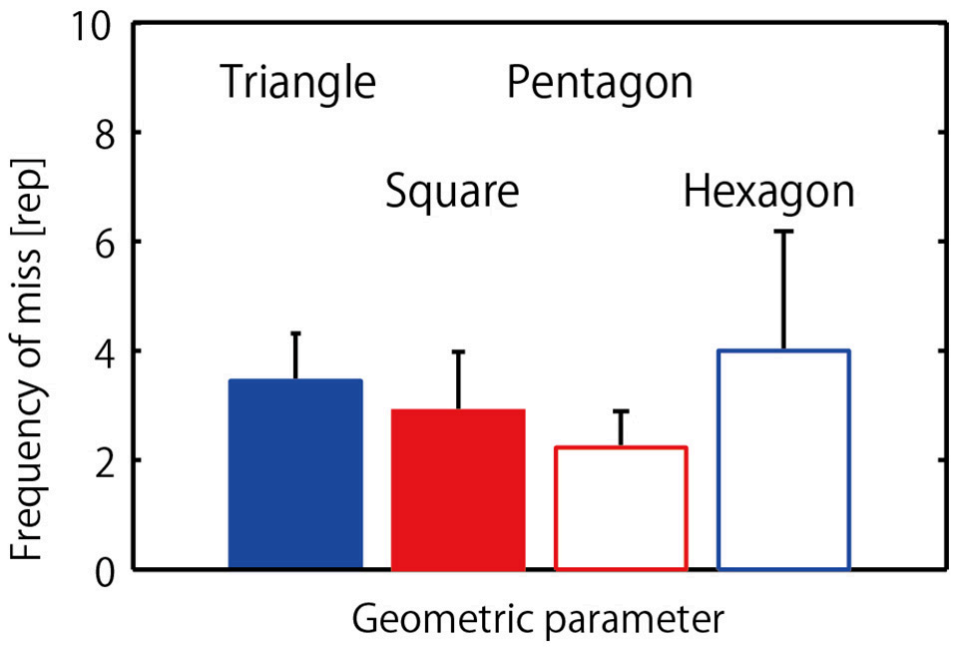
**Frequency of misses (collisions) that occurred in each condition**.

Figure 4 displays mean and the standard deviation of the frequency of misses or unsuccessfully jumps (participant collisions) that occurred prior to achieving a successfully 20-jump sequence. The one-way repeated measure ANOVA revealed no significant difference between the four different geometrical hoop conditions, *F*_(3, 24)_ = 0.52, *p* = 0.67, η^2^ = 0.26.

#### 2.2.2. (A)symmetry in temporal coordination

**Figure 5:**
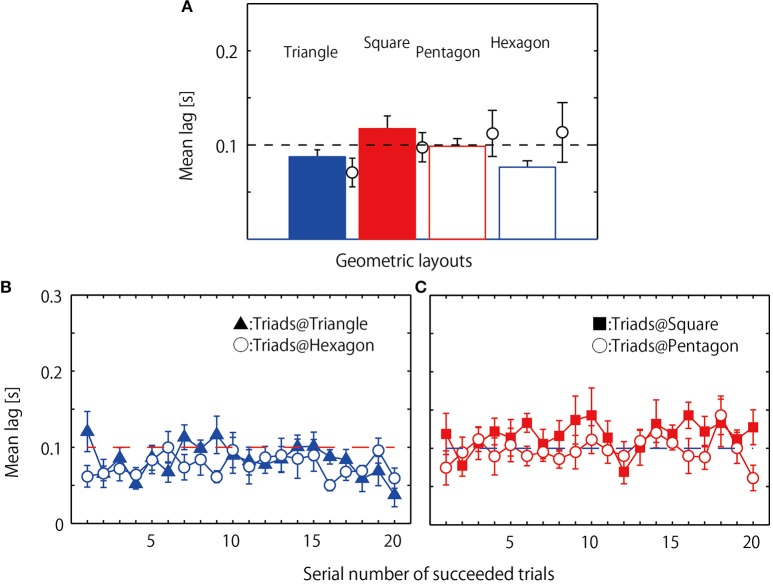
**Coordination lag observed during successful jumping trials**. Mean of eight triads each averaged over 20 successful trials. **(A)** Circles and error bars indicate mean and standard error of one excluded triad. **(B)** Mean lag averaged over eight triads separately for each of 20 successful trials observed in the two even geometrical conditions; filled triangle indicates mean and standard errors in triangle condition and open circle indicates those for hexagon condition. **(C)** Mean lag averaged over eight triads separately for each of 20 successful trials observed in the two asymmetrical geometrical conditions; filled square: square condition, open circle: pentagon condition.

The overall mean coordination lag of the two followers' relative to the leader is displayed in Figure 5A. The statistical analysis revealed a significant main effect of geometrical hoop condition, *F*_(3, 24)_ = 4.497, *p* = 0.012, η^2^ = 0.75, with *post-hoc* analysis indicating that the lag in the square condition was significantly longer than the lag observed for the triangle (*p* = 0.019) and hexagon (*p* = 0.019) conditions. This same analysis was also performed after excluding one triad, with the corresponding mean data superimposed in Figure 5A using open black circles. This triad (Triad H in Table 2) was excluded from this and subsequent analysis because their overall task performance was much poorer than the other triads and, moreover, because the patterning of the jumping behavior exhibited was qualitatively different from the other triads (see below for details on the performance of this triad). The analysis of these data also resulted in a main effect of geometrical hoop condition, *F*_(3, 21)_ = 6.377, *p* = 0.003, η^2^ = 0.955, but this time with the coordination for both the square and pentagon conditions being significantly longer than that observed for the triangle and hexagon conditions (square-triangle: *p* = 0.024; square-hexagon: *p* = 0.024; pentagon-triangle: *p* = 0.052; pentagon-hexagon: *p* = 0.041).

Figures 5B,C shows the mean lag of the eight triads retained for analysis (i.e., excluding the triad depicted by the circle means in Figure 5A) calculated separately for the 20 successful jumps for the four hoop conditions. Consistent with the results of overall mean lag presented above, a geometrical condition (triangle, square, pentagon and hexagon) by 20 jump events two way repeated measures ANOVA revealed a significant main effect of geometrical hoop condition, *F*_(3, 21)_ = 6.390, *p* = 0.003; η^2^ = 0.96, with the lags for the square and pentagon conditions being consistently longer than those for the triangle and hexagon conditions (*post-hoc* Benjamin-Hochberg analysis, *p* < 0.001). There was no main effect of jump event, *F*_(19, 133)_ = 1.130, *p* = 0.330; η^2^ = 0.139, nor an interaction between geometric condition and jump event *F*_(57, 399)_ = 1.217, *p* = 0.15 η^2^ = 0.148.

**Figure 6:**
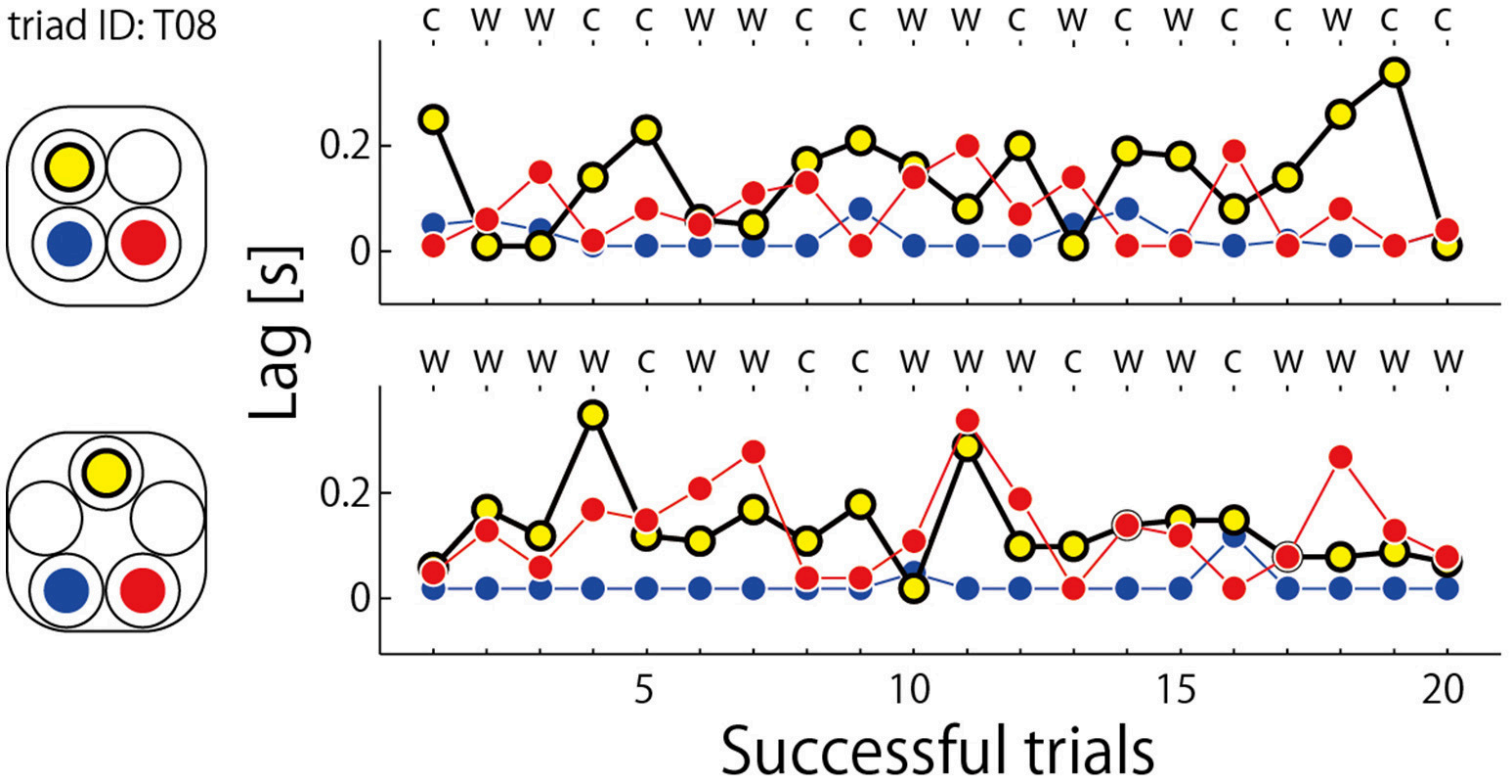
**Time series of lag relative to the onset of leader's jump (=0 s) for the exceptional triad (ID: T08, Triad H in Table **2**)**. Initial position of each member of the triad was illustrated in the left panel. The letter on each abscissa scale indicates rotation direction of the triad; w, clockwise; c, counterclockwise.

The data presented in Figure 6 displays the jump lags observed for the exceptional triad identified above and in Figure 5A. For this triad, the participant who was assigned to the blue hoop starting location always led the other two participants irrespective of rotation direction or geometric constraint. Although defining a single leader across conditions is a possible strategy for achieving coordinated jumping, no other triad exhibited a consistently stable pattern of participant leading and there is no reason why such single leader dominance should occur in this manner for the two asymmetric conditions unless some a-prior “decision” is made as to who will lead a given jumping sequence or set of jumping sequences. Indeed, the participant in the blue starting location was less likely to lead in all other triads in the square and pentagon conditions (see **Figure 8**). They jumped eight and four additional trials due to coordination misses in the square and pentagon conditions respectively, whereas mean ± SD of misses averaged over the eight triads in each of two conditions was 2.250 ± 1.982 for square and 2.000 ± 1.604 for pentagon. Thus, the triad that included a member who can jump into the space occupied by others show poorer performance, and this manner he/she always took was idiosyncratic relative to others.

**Figure 7:**
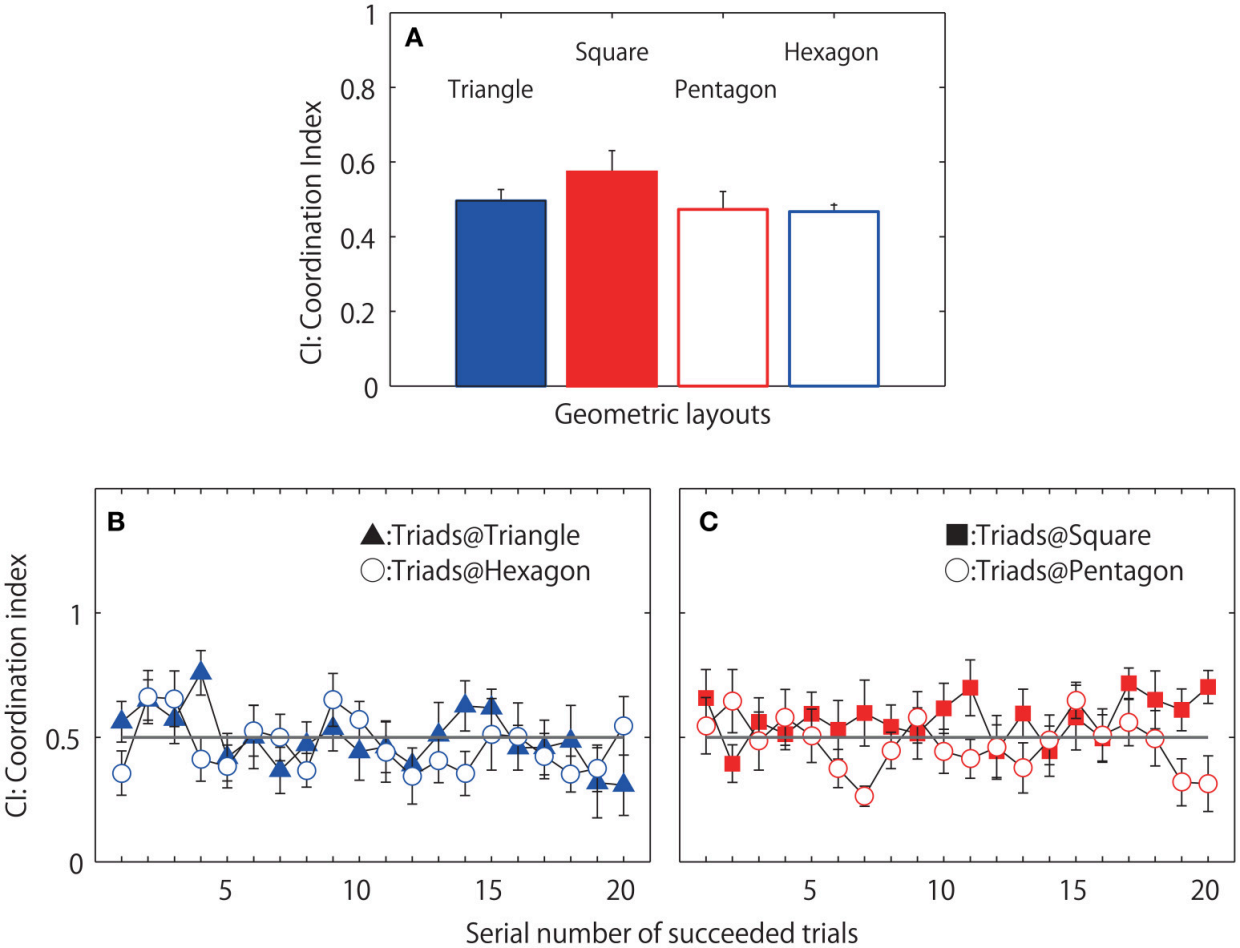
**Coordination index (CI) observed during successful jumping trials**. Mean of eight triads each averaged over 20 successful trials **(A)**. Mean CI averaged over eight triads separately for each of 20 successful trials observed in two even geometrical conditions, filled triangle indicates mean, and standard errors in triangle condition and open circle indicates those for hexagon condition **(B)** and the CI observed in two asymmetrical geometrical condition; filled square: square condition, open circle: pentagon condition **(C)**.

**Figure 8:**
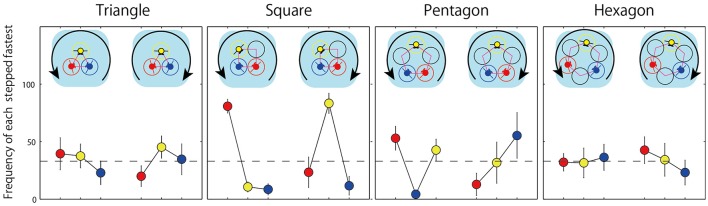
**Frequency of the case in which each member jumped earlier than others observed in four conditions**. Each contains both rotation direction of counterclockwise and clockwise.

Figures 7B,C displays the mean CI of the eight triads calculated separately for the 20 successful jump events. The two-way geometrical condition (triangle, square, pentagon and hexagon) by 20 jump events ANOVA revealed a significant main effect of jump event [*F*_(19, 133)_ = 1.791, *p* = 0.030; η^2^ = 0.506] (Figure 7A displays the mean and standard error of 20 jump events). However, *Post-hoc* analysis indicate no significant difference between jump events. There was no main effect of geometrical condition [*F*_(3, 21)_ = 1.210, *p* = 0.330; η^2^ = 0.416] nor an interaction [*F*_(57, 399)_ = 1.0831, *p* = 0.326; η^2^ = 0.393], which in contrast to expectations initially suggested an equal preference for 1-leader/2-follower and 2-leader/1-follower CI relationships across conditions (although see below and Figure 8 for more details).

Finally, for each triad we identified the participant that jumped faster than the other two participants in each of the four geometric conditions (i.e., the leader vs. the followers) to determine the frequency that a particular participant position was the leader as a function of jump direction (i.e., clockwise or counterclockwise). These “leader/fastest jumper” frequencies are displayed in Figure 8. One way-ANOVAs were employed to compare these frequency counts for each rotation direction in each geometrical condition. Consistent with isotropy subgroup expectations detailed above and listed in Table 1, the results revealed no significant effect of a members location in two symmetrical conditions (triangle counterclockwise: *F*_(2, 14)_ = 0.675, *p* = 0.525, η^2^ = 0.311; triangle clockwise: *F*_(2, 8)_ = 0.917, *p* = 0.438, η^2^ = 0.479; hexagon counterclockwise: *F*_(2, 14)_ = 0.040, *p* = 0.961, η^2^ = 0.076; hexagon clockwise: *F*_(2, 10)_ = 0.420, *p* = 0.668, η^2^ = 0.290), but a significant effect in almost all of the asymmetrical conditions (square counterclockwise: *F*_(2, 14)_ = 42.737, *p* = 0.000, η^2^ = 2.471; square clockwise: *F*_(2, 8)_ = 10.682, *p* = 0.006, η^2^ = 1.634; pentagon counterclockwise: *F*_(2, 14)_ = 6.601, *p* = 0.001, η^2^ = 0.972) excluding the case of clockwise rotation in pentagon condition [*F*_(2, 8)_ = 1.121, *p* = 0.372, η^2^ = 0.530]. Thus, in the asymmetrical square and pentagon conditions, the participant next to an open jump location in the direction of the previously jumped rotation jumped faster than the other jumpers in 60(in pentagon)-80(in square)% of trials they coordinated successfully. This asymmetric preference was not observed in the triangle and hexagon conditions, with a more symmetric (non preference) leader/follower relationship exhibited across jump events. Normative (symmetric) probability of first jumper (leader) can be postulated as 33%(1/3) (highlighted with broken line in Figure 8).

## 3. Discussion

The current study investigated whether the patterns of behavioral coordination during a cooperative, three-person (triadic) jumping task were defined by the (a)symmetries of an participant-hoop configuration. Of particular concern, was the degree to which the symmetries of the actor's jumping direction dynamics and the temporal lead/lag relationship (i.e., leader/follower role) between actors was a functional reflection of the symmetry group(s) that defined the participant-hoop configuration. Here we discuss the degree to which the current findings support these symmetry (and group theory) based expectations, first with regards to the triads' jump direction decisions and then with regards to the degree to which the observed (a)symmetries in the temporal lead/lag and leader/follower relationship of co-actors was consistent with the hoop-triad isotropy subgroups defined in Table 1.

### 3.1. Asymmetry in jump direction decision

As noted above, it is important to appreciate that the symmetry between counterclockwise/clockwise jumping needed to be collectively broken on each and every trial in order for the triads to complete a successful jump. Indeed, the chance of a triad achieving a single jump successfully was only equal to 2/2^3^ = 2/8 = 1/4 or 25% if each participant in a triad were to randomly choose a jumping direction. Accordingly, participants in a triad were required to make a “collective” decision about which direction to jump, to the left (clockwise jump) or to the right (counterclockwise jump), on any given jumping trial. This was true for all hoop conditions, in that for all conditions a collision (failed trial) would result if any one participant decided to jump in a direction different from their co-participants. Thus, all of the geometric hoop conditions entailed the same two action possibilities on any given jump, with the potentiality of clockwise and counterclockwise jumping being equivalent. Consistent with our expectations, participants exhibited an asymmetric preference in jumping direction within and across jumping sequences. That is, participants “broke” the symmetry between clockwise and counterclockwise jumping. The equivalence of these two action possibilities in all of the geometric hoop conditions was also reflected by the fact that no difference in jump-to-jump decision dynamics were observed for the different geometric hoop conditions. However, in contrast to expectations, triads did not show an equal or symmetric preference for each jump direction across triads and/or jumping sequences. Rather, triads exhibited a strong preference for the counterclockwise direction (i.e., 70% of successful coordinated jumps were counterclockwise) in all four geometrical hoop conditions.

Interestingly, most of the participants reported retrospectively that subtle changes in the knee extension and flexion movements of their co-actors often indicated which direction they should jump on any given trial. Thus, the decision about which direction to jump could be understood as having occurred spontaneously - *spontaneous symmetry break* - on any given jump, a result of the subtle, yet coordinated fluctuations in the movement dynamics of the triad. Intuitively, one would expect that observing each other's knee movements would not only support the efficacy of the participants' decision about which direction to jump, but would also support the synchronization of the triads preparatory movements and, thus, the ability of the participants to collectively jump in time with the metronome signal. However, although movement fluctuations and the visual coupling between the oscillatory jumping movements of the actors may account for the jump direction chosen on each individual trial, as well as the extremely short lag between members' jumping actions (Figure 5), it still does not account for the overall preference for counterclockwise jumping over clockwise jumping.

Perhaps the most likely reason for this counterclockwise bias was a physiological factor, such as hand or foot dominance, with this a-priori biomechanical asymmetry operating as an *explicit symmetry-breaking* factor on the behavioral organization of the triad. With regards to overall task success, however, a persistent break in the symmetry of counterclockwise vs. clockwise jumping was the most effective strategy for completing a successful 20-jump sequence. That is, always jumping to the left, rather than to the right (or vice versa), best supported the continuous jumping behavior of the participants by minimizing the decision function to only one possibility (thereby reducing the actor's cognitive load and/or need for a strong perceptual attunement to the movements dynamics of others). Accordingly, it is possible that even if hand or foot dominance was not the reason for the counterclockwise bias, other non obvious task asymmetries such as the direction of the auditory metronome tone or experimenter position, may have operated to break the symmetry of the action space. Note, however, that these or other a-priori breaks in symmetry would only need to influence performance on the first jump within a sequence, with this past jumping action then operating as a symmetry-breaking factor on future jumps within the same sequence (or even across sequences), further increasing the preference of the counterclockwise direction relative to the clockwise direction.

### 3.2. Relational symmetry of behavioral coordination and the actor-environment task space

The results revealed that the difference in participant-hoop configuration for the symmetric (3- and 6-hoop) and asymmetric (4- and 5-hoop) conditions influenced the patterning of the temporal coordination between participants in two ways. As illustrated in Figure 5, the first effect was that the overall average lag between jumpers was significantly longer in asymmetrical conditions compared to the symmetrical conditions. However, it is worth noting that the overall average temporal lag between participants was very small for both types of conditions, with the lag for the symmetrical conditions approximately equal to zero and approximate equal to 0.1 s for the asymmetrical conditions. Indeed, the latter lag is still very short compared to standard estimates of human whole body reaction times (0.358 ± 0.600 s in 20 years. for Japanese male and 0.410 ± 0.280 s in 20 years. for female; Japan Industrial Safety and Health Association). As discussed above, these short latencies suggest that in both the symmetrical and asymmetrical conditions each member of triad was able to successful predict when and in what direction the other members of the triad were intending to jump (again, likely due to the detection of knee flexion-extension kinematics).

The second and much more important finding related to the symmetries that defined the frequency with which each actor led (or followed/lagged) the jumping action during successful 20 jump sequences. As illustrated in Figure 8, the frequency of participant role (i.e., leader/first jumper vs. follower/lagged jumper) was invariant for the symmetrical hoop conditions, with each actor equally likely to jump first. In contrast for the asymmetrical conditions, there was a strong asymmetry in the frequency of actor role, with a greater magnitude of invariance in terms of the role adopted by a given actor across jumping events - that is, one actor adopted the role of leader or follower more often than the other two actors. This latter asymmetry in actor role is particularly clear in the square condition, but is also discernible in the pentagon condition.

Of course, the significance of this latter finding is that its is consisted with the hypothesis *that the symmetry (asymmetry) of triad behavior would correspond to the symmetry (asymmetry) of the highest order isotropy subgroup of the participant-hoop configuration*. For the triangle and hexagon conditions, the highest order, *D*_3_, isotropy subgroup of the participant-hoop configuration was reflected by the fact that each actor was equally likely to emerge as the leader on any given jump event. Moreover, this suggests that the emergence of the lead jumper on any given jump was the result of a spontaneous symmetry break (i.e., could have resulted spontaneously from small temporal fluctuations in actor movement onset/offset times). As already noted, this is consistent with the findings displayed in Figure 8, with each actor equally likely to jump first on any given jump trial during the triangle and hexagon conditions. In contrast, for the two asymmetrical conditions, the highest order isotropy subgroup for the square and pentagon conditions was *D*_1_ (or *Z*_2_,). This reflected the (explicitly broken) asymmetry in the jumping DoF available to each actor in these two conditions. The corresponding symmetry or group theoretic prediction was that only two actors should be permutable or interchangeable within the task context. That is, one actor should consistently behave differently from the other two. Consistent with this predicted, a more predictable pattern of “leaders” and “followers” was observed for the square and pentagon conditions compared to the triangle and hexagon conditions (i.e., less leader-follower interchange; see Figure 8). More specifically, the participant or participants who had open locations next to them in the direction jumped previously, tended to jump first (leading) compared to the other actor or actors. For instance, in the square condition the “red” actor in Figure 8 led more often during clockwise jumps, whereas the “yellow” actor led more often during counterclockwise jumps. Similarly, for the pentagon condition the “blue” and “yellow” actors were more likely to lead/jump first compared to the “red” actor. Note the latter, pentagon 2-to-1 symmetry and the former, square 1-to-2 symmetry are both entailed by *D*_1_ and *Z*_2_.

Finally, it is important to note that the participant-hoop isotropy subgroups defined in Table 1 (in method section) represent the set of possible behavioral modes that could have occurred, such that lower order coordination patterns were still stable and could have emerged (recall that anti-phase coordination is still a stable pattern of rhythmic inter-limb coordination even though in-phase coordination is the more symmetric pattern Kelso, 1984, 1995). The implication for the jumping task investigated here, is that during the triangle and hexagon conditions triads could have adopted the same pattern of behavior they exhibited in the square and pentagon conditions (i.e., *D*_1_ or *Z*_2_,), as well as a cyclic leader/follow pattern (*Z*_3_) or even a fixed pattern of behavioral roles (i.e., an I or Identify pattern). Of course, the latter identity pattern was the only other option available to triads in the square and pentagon conditions and would correspond to each actor adopting a fixed, asymmetric role (i.e., leader, second, third jumper) across jump events (to some extend this may have defined clockwise pentagon jumps; see Figure 8). As noted in the introduction, however, self-organized dynamical systems typically (or more often) converge on the most symmetric pattern of behavior possible within a given task context (e.g., in-phase in laboratory joint action task: Schmidt et al., 1990; Richardson et al., 2007b; anti-phase in one-on-one competing action: Kijima et al., 2012; Okumura et al., 2012), with such states being more stable in the absence of further symmetry breaking factors. Indeed, for the present task, the emergence of lower order isotropy subgroup symmetries would have required an explicit or induced symmetry break on the part of the participants (Richardson et al., 2016). For instance, the participants would have needed to explicitly communicate or agree on an order or permutation pattern of actor role. The lower-order behavioral modes of coordination could have also been explicitly induced by employing visual information to form a shared task representation of each actors intentional state (Sebanz et al., 2003, 2005, 2006b). This may well have been what resulted in the qualitatively different behavior of the excluded pair shown in Figure 6. An interesting question for future research, is whether such cognitive or representational forms of explicit symmetry breaking might be directly specified and (cognitively) understood via the perception of shared task affordances (Sebanz et al., 2006a; Richardson et al., 2007a; Marsh et al., 2009).

## 4. Conclusion

In conclusion, the results of the current study reveal that the geometrical (a)symmetry of an actor-environment task space determines the (a)symmetry of the behavior coordination that can emerge. The current study also demonstrates how the formal language of symmetry, namely group theory, can be employed to understand and define the patterns of behavioral coordination that are possible and likely to occur within the given (multi-) agent-environmental task context. The extended implication is that the principles of symmetry and symmetry-breaking can provide a fundamental and highly generalizable theory for understanding and predicting the stable patterns of multi-agent coordination and social activity, one that places a theoretical account of psychological perceptual-motor behavior, as well as cognitive decision making within the formal principles that shape and constrain all biological and natural systems (Richardson et al., 2016).

## Author contributions

AK, HS, MO, and YY contributed to the conception and design of the work. AK, MO, and YY contributed to the data acquisition. AK HS, MO, YY, and MR contributed to the data analysis and interpretation of data. AK, HS, YY, and MR work for drafting and HS, MO, YY, and MR revised the manuscript critically for important intellectual content. All authors contributed in final approval of the version to be published. AK and MR are accountable for all aspects of the work in ensuring that questions related to the accuracy or integrity of any part of the work are appropriately investigated and resolved.

## Funding

This work was supported by JSPS KAKENHI Grant Number JP23500711 and JP25390147. The research was also supported, in part, by an award from the National Institutes of Health, R01GM105045.

### Conflict of interest statement

The authors declare that the research was conducted in the absence of any commercial or financial relationships that could be construed as a potential conflict of interest.
